# Impact of Childhood Cancer on Family Functioning and Family Quality of Life in the Western Region of Saudi Arabia

**DOI:** 10.1155/2024/6667978

**Published:** 2024-01-19

**Authors:** Tagwa Yousif Omer

**Affiliations:** College of Nursing, King Saud Bin Abdulaziz University for Health Sciences, P.O. Box 9515, Jeddah 21423, Saudi Arabia

## Abstract

**Background:**

Childhood cancer affects families and friends and causes lifestyle changes that become overwhelming for them. Childhood cancer may cause decreased physical, emotional, and social health-related quality of life (QOL). Childhood cancer may cause strain on the financial status of the family and shape their coping strategy to the disease. The extent of the impact of childhood cancer on families is associated with several demographic characteristics of the family such as diagnosis, phase of treatment, and parent's educational level, employment, and marital status of the parents.

**Objectives:**

The objective of this study was to explore the impact of childhood cancer on family functioning and family quality of life (QOL) in the Western Region of Saudi Arabia.

**Methods:**

This study was a quantitative, randomized, cross-sectional study. 187 participants were randomly selected from the population of parents whose children have cancer and treated at Princess Noorah Oncology Center in King Abdulaziz Medical City, Jeddah. A survey was used to collect data for this study. Healthcare and social systems may have to consider the impact of childhood cancer in the care plans of the patients.

**Result:**

Leukemia represents the highest disease prevalence followed by brain tumor. The highest score of the impact on the family survey was familial social concerns domains followed by financial burden with mean scores of 3.59 (98.8%) and 3.56 (98.0%), respectively. Then, mastery domain mean score is 3.43 (85.8%) and finally personal strain with mean score of 3.21 (980.3%). The QOL of the family results indicated that the highest was physical/material well-being with mean score of 3.84 (76.8%) and family interaction with mean score of 3.82 (76.4%), followed by emotional well-being with mean score of 3.54 (70.8%) and parenting with mean score of 3.53 (70.6.%). Significant differences were found between the overall scales of QOL and the scale of impact on the family and some demographic characteristics of children and their parents.

**Conclusions:**

Childhood cancer has a substantial effect on family functioning and the family's QOL. In addition, both were significantly associated with some demographic characteristics of the child and his parents.

## 1. Background

The incidence of childhood cancer tends to increase worldwide. More than 400,000 children aged birth to 19 years are diagnosed with cancer each year around the world [1]. Diagnosis of cancer for any family member affects the whole family and friends and causes lifestyle changes that become stressful and overwhelming for the family. Childhood cancer has a high association with the domains of impact on family [2]. Families that have a child diagnosed with cancer may decrease physical, emotional, and social health-related QOL of these families [2, 3].

Several studies have proven that childhood cancer is associated with family functioning domains such as cohesion, expressiveness/communication, conflict, adaptability, and support [4–8].

A study conducted in Turkey identified that children with cancer and their families experienced major psychosocial and financial problems [9]. Furthermore, 62% of families need financial support and 49% of families borrowed money or have loans. In addition, 69% of families experienced difficulties to care for the other healthy siblings and 43% of mothers experienced severe psychological problem during and after treatment [9].

In Sweden, a qualitative study revealed two main themes. The first is unfamiliarity and frightening situation during treatment, which related to initial reactions to uncontrollable situation, adjustment to situations, and focus on supporting the sick child during treatment. The second was emotional struggles after the end of curative treatment which related to transitioning back to life as it was before the diagnosis, emotional scars, uncontrollable fears and worries about the cancer disease, and new perspective of life after the treatment [10].

A phenomenological study of parents' experience for caring of children with cancer identified challenges that parents face, including anxiety of the death of their children, inability to respond to the questions of their children, inability to have an appropriate behavior while confronting the children angry, suffering of treatment side effects in their children, the pressure of economic, social, and psychological burden on family, lack of time, the impact of spiritual support, and the influence on the relationship between parents [11]. Another study done in Jordan found out that there were significantly higher stress scores in parents who have child with cancer than those with no seriously ill child [12].

Caregivers of children with cancer presented with burden, mostly isolation, disappointment, and compromised aspects of QOL. The scores of QOL of caregivers of children with cancer were lower than the control group in the eight domains: physical functioning, role physical, bodily pain, general health perception, vitality, social role functioning, emotional role functioning, and mental health [13]. A systematic review on impact of a cancer diagnosis on family caregivers revealed that there was negative impact on caregivers who experienced high distress, diminished quality of life (QOL) and reported moderate to high fear of cancer recurrence [14].

There was no enough literature on the experience of families who have children with cancer in Saudi Arabia. The aim of this study is to explore the impact of childhood cancer on family functioning and family QOL in the Western Region of Saudi Arabia.

### 1.1. Objectives of the Study

1. To assess the impact of childhood cancer on family functioning
2. To determine the QOL of family whose children have cancer
3. To identify the correlation of the demographic characteristics of patients and their parents and impact of childhood cancer and family QOL

## 2. Methodology

### 2.1. Study Design

This study was a quantitative, randomized, cross-sectional study. Participants were randomly selected from the population of parents whose children have cancer and treated at Princess Noorah Oncology Center in King Abdulaziz Medical City, Jeddah.

### 2.2. Setting

This study was conducted at an oncology center in a 750-bed tertiary care hospital in the city of Jeddah, Saudi Arabia. The oncology center is one of the biggest centers for cancer treatment in the Western Region of Saudi Arabia, and it includes adult and pediatric oncology services and has outpatient, inpatient, and radiation therapy services. The pediatric hematology oncology inpatient service currently has 35 beds including pediatric BMT.

### 2.3. Sampling Procedures

A simple radom sample was used in this study, and all randomly selected children under 18 years of age were invited with their parents to participate. According to the statistics in PNOC, there are 370 oncology pediatric patients followed up and treated in the center. The sample size was calculated using the modified Cochran formula for sample size calculation in smaller populations [15]. The estimated sample size will be *n* = 187 participants. The 370 were put in one list. Randomization was carried out by randomly selecting each other patient from the list through the computer. The selected participants were contacted by the researcher and invited to fill the questionnaire after signing an informed consent.

Several strategies were used by the researcher to avoid sampling bias. First is the definition of the target population and the sampling frame before data collection. Second is the randomization of sample. Third is the follow-up of nonrespondents to ensure their voluntary participation.

### 2.4. Data Collection

Three tools were administered to the parents of a child less than 18 years old:

(1) Sociodemographic and health-related survey that is developed by the researcher after literature review [12, 13, 16]: This part includes patients and parents demographic characteristics.
(2) Impact on the family scale which is a 24-item questionnaire answered on a 4-point Likert-type scale (strongly agree–strongly disagree): This scale was designed to measure the family impact of chronic medical illnesses in four dimensions [17, 18].
  - Financial burden (changes in the financial status of the family): It consists of 4 items.
  - Familial/social impact concerns level of disruption of interaction within the family unit and outside the family: It consists of 9 items.
  - Personal strain-psychological burden experienced by caregiver of the child with cancer: It consists of 6 items.
  - Mastery-coping strategy employed by the family: It consists of 5 items.
Cronbach alpha reliabilities are 0.72, 0.86, 0.81, and 0.60, respectively, and the total score reliability is 0.88.
(3) The family QOL (FQOL) scale [19]: This scale is a 21-item inventory rate on a 5-point Likert-type scale (very satisfied–very dissatisfied). Its purpose is to measure the family QOL under four subscale domains:
  - Family interaction (6 items)
  - Parenting (6 items)
  - Emotional well-being (4 items)
  - Physical/material well-being (5 items)

Cronbach reliabilities for FQOL subscales are 0.75, 0.71, 0.76, and 0.77, respectively. The total score reliability is 0.88.

### 2.5. Data Analysis

Data collected were stored, prepared, and coded in Excel sheets prior to the data analysis process. SPSS software, version 25, was used to analyse the data. Categorical variables were presented in frequencies and percentages, and quantitative continuous variables were described by measures of descriptive statistics including mean scores and SD. Significant differences in study scales were examined using the *t* test for independent groups and ANOVA. Correlation analysis was conducted to examine the strength and direction of the relations between scales components. A *p* value of less than or equal 0.05 was considered significant.

### 2.6. Ethical Considerations

Approval for this study was obtained from the Research Committee of the College of Nursing as well as from Human Subject Board (IRB) of King Abdulla International Medical Research Center. All participants were invited to participate by invitation letter and received informed consent form with the questionnaire ensuring that the participation is voluntary. The invitation letter contained the purpose of the study, research procedure, and a guarantee to maintain anonymity and confidentiality of the information. No names of participants and medical ID numbers were disclosed in any questionnaire. Collected data were kept in a secured safe. Only the PI has an access to it.

## 3. Results

### 3.1. Demographic Characteristics of Participants

A total number of 187 families of a child with cancer were included in this study. Data regarding the demographic characteristics were collected for both children with cancer and their parents. Demographic data of the children with cancer revealed that the mean age is 6.3 years with 3.6 SD. Approximately half of the patients were males and half were females. Leukemia represents the highest disease prevalence (41.6%), followed by brain tumor (25.3%) of all types of childhood cancer. The mean of time since diagnosis was 2.0 years (SD 1.4), and most of the children with cancer are still on treatment, 154 (86.4%).  Table 1 shows the demographic data of the children with cancer.

The mean ages of mothers and fathers are 35.5 and 39.7 years, respectively. About 90% of parents were married at time of data collection, while 10% of them were divorced. All parents were educated with at least primary level, and 43.3% of fathers had a university degree, compared to 27.0% of mothers. Most fathers were employed, while the majority of mothers were unemployed. The mean number of children in family was 3.9 children. Characteristics of parents are presented in Table 2.

### 3.2. Impact of Childhood Cancer on the Family

The mean and percentage of agreement for each domain of the impact on the family survey were calculated and arranged from high to low mean scores. Mean scores of familial social concerns (level of disruption of interaction within the family and outside the family) and financial burden (changes in the financial status of the family) domains are 3.59 (89.8%) and 3.56 (89.0%), respectively. Then, mean score of mastery (coping strategy employed by the family) is 3.43 (85.8%), and finally, mean score of personal strain (psychological burden experienced by care giver of the child with cancer) is 3.21 (80.3%). Results are presented in Table 3.

Items within each domain of the impact of childhood cancer on the family survey were analysed. The mean and SD for each item were calculated and then arranged from high to low mean scores. For the financial burden domain, the highest item's mean is “additional income is needed to cover medical expenses” with mean score of 3.64 and the least item's mean in this domain is “time is lost from work because of hospital appointments” with mean score of 3.54. For the familial social concern domain, the highest item's mean was “because of the illness, we are not able to travel out of the city” with mean score of 3.70 and the lowest item's mean is “people in the neighborhood treat us specially because of my child's illness” with mean score of 3.48. The third domain is personal strain. The highest rated item's mean was “nobody understands the burden I carry” with mean score of 3.52 and the least scored item is “travelling to the hospital is a strain on me” with mean score of 2.40 where participants agreed that this is not a challenge for them. The final domain is mastery, the highest item was “learning to manage my child's illness has made me feel better about myself” with mean score of 3.55 and the lowest item was “my relatives have been understanding and helpful with my child” with mean score of 3.32.  Table 4 shows the results of the impact on family domains.

### 3.3. Quality of Life of the Families with Childhood Cancer

Family QOL domains' mean and percentage of agreement were also calculated and arranged. The five domains were ranked from the highest mean to the lowest mean. The highest mean was physical/material well-being with mean score of 3.84 (76.8%) and then family interaction with mean score of 3.82 (76.4%), followed by emotional well-being with mean score of 3.54 (70.8%), and the lowest is parenting with mean score of 3.53 (70.6%). Data are presented in Table 5.

Each of the quality of life domain items was ranked from the highest to lowest mean. In the physical/material well-being domain, the highest item's mean score is “my family feels safe at home, work, school, and in our neighborhood” with mean score of 4.29 (SD 0.93) and the lowest item is “my family gets dental care when needed” with mean score of 3.23 (SD 0.91). In the family interaction domain, the highest item's mean score is “my family is able to handle life's ups and downs” with mean score of 3.90 (SD 0.89) and the lowest item's mean score is “my family enjoys spending time together” with mean score of 3.73 (SD 0.73). The highest item's mean score for emotional well-being domain is “my family members have some time to pursue their own interests” with mean score of 3.61 (SD 0.95) and the lowest item's mean score is “my family members have friends or others who provide support” with mean score of 3.47 (SD 0.92). Regarding the parenting domain, the highest mean for the item is “family members help the children learn to be independent” with mean score of 3.66 (SD 0.80) and the lowest mean for the item is “adults in my family have time to take care of the individual needs of every child” with mean score of 3.45 (SD 0.10). Data on family QOL subscale are presented in Table 6.

### 3.4. Correlation between Overall Scores of Impact of Family Domains and Demographic Characteristics of Participants

The strength of the relationship between demographic characteristics and overall score of impacts on family domains was tested using correlation coefficient. Results revealed significant correlations between the impact of family domains and most of the demographic characteristics except financial burden with both mother and father employment status (*p* > 0.05) and personal strain with fathers' age, employment status, and number of siblings (*p* > 0.05). The mastery domain is also significantly correlated with all demographic characteristics except time since diagnosis, fathers' age, and fathers' employment status (*p* > 0.05). Data are presented in Table 7.

### 3.5. Correlation between Overall Scores of Quality of Life Domains and the Demographic Characteristics of Participants

The strength of the relationship between demographic characteristics and the families' quality of life domains was tested using correlation coefficient. Results indicated that some of the quality of life domains were significantly correlated with some demographic characteristics of the participants. Family interaction did not correlate with any of the demographic characteristics. Parenting domain has a positive correlation with all demographics except age and gender (*p* < 0.05). Emotional well-being domain has a positive correlation with the phase of treatment and the mothers' employment status (*p* < 0.05). Physical/material well-being domain had a positive correlation with age and phase of treatment (*p* < 0.05). Data on correlation are presented in Table 8.

The correlation matrix of quality of life domains and impact in family domains is shown in Table 9. The results reported significant correlations between some of the domains of scales. Family interaction was significantly and positively correlated with financial burden only, while parenting was significantly and negatively correlated with all domains of impact on family factors except the financial burden domain. Physical well-being domain was significantly and positively correlated with all four domains of impact on life domains.

## 4. Discussion

This study aims to explore the impact of childhood cancer on family functioning and family QOL. Childhood cancer is one of the very stressful and life-changing experiences for families and has a profound impact on the family. 187 families of children with cancer were included in this study. Results indicated that leukemia and brain tumor represent the highest prevalence of cancer types. This result is congruent with the result of International Agency for Research on Cancer [20] and locally with the report from the Ministry of Health (MOH) which indicated there are 12 types of children's cancers in Saudi Arabia and leukemia and brain cancers are among the most common childhood cancers followed by all other types of childhood cancer [21]. The mean age of children involved is supported by [3], who found that the clinical features of childhood cancers are mostly manifested and diagnosed at an early age (within five years) of the children.

Families of children with cancer are faced with several issues that affect the family's QOL. Despite this, research studies on the impact of childhood cancer on the families have not been conducted in Saudi Arabia. Results of this study indicated that the four domains of the QOL scale, physical well-being, family interaction, emotional well-being, and parenting, were affected. Other studies had found the same results; parents who have children with cancer reported physical symptoms, such as fatigue (68%) and difficulty sleeping (51%), and parents reported somatic disorders more often if children were ill for more than 3 years [22]. Similar results found that nearly 50% of their study population experienced low QOL, and this low score was significantly associated with level of parent's education, lower socioeconomic status, prolonged treatment duration, and increasing cost of treatment [23, 24]. A comparison study performed to assess the QOL of parents of children diagnosed with cancer compared to parents of children with minor ailments found that the QOL of parents of childhood cancer was significantly impaired in psychological domain, social relationship domain, and environmental domain [13].

The diagnosis of childhood cancer can pose substantial challenges to families. The result of this study revealed that childhood cancer has a great impact on the family. A study done in Bangladesh, using the same survey that was used in this study, revealed that all four domains were affected with different sequences, this study result [3]. The Bangladesh family achieved the highest score in mastery as their top-performing aspect, with a rating of 3.63. Following closely were financial burden, reflecting changes in financial status (3.33); personal strain, indicating the psychological burden experienced (3.28); and lastly, social impact, representing the level of disruption of interaction within and outside the family (3.2) [3]. Another study revealed that parents of children who suffered from cancer struggled with various problems [22]. The first is the financial problem, where almost half of the participating families (44%) believed that their financial situation worsened with the child's cancer to a moderate extent and 39% believed it worsened to a large extent. The second is the psychological problem, where 20% of parents were devastated, 75% of the parent felt anxiety, and 35% of the parents received support from their own families. The third is the family relationship problem, where families indicated that the child's disease did not change the relationship within the family (41%); it strengthened family ties in 32% and the family relations deteriorated in 27% [22].

The results of this study identified significant differences between the overall scales of QOL and the scale of impact on the family and some demographic characteristics of children and their parents. Family functioning and the appraisal of the cancer diagnosis proved to be related to cancer-related emotions of patients and their family members and QOL after the diagnosis of the cancer [6]. A study concluded that the occurrence of problems for parents of children suffering from cancer had a significant negative correlation with both the age of the parents and the level of education [22]. In addition, parents with financial problems more often had their children ill for a long time [22]. These results are congruent with the study conducted in Bangladesh, where the difference in impact on family score was significantly correlated to the father's occupation, type and duration of cancer, and treatment cost [3]. Family relationships were also impaired when diagnosed with leukemia/lymphoma compared to solid tumor [16].

A study conducted in South India revealed that QOL was significantly associated with the age of the child, parents' level of education, and the type of parents' work [24]. The results also found that difference in QOL values was significant for lower socioeconomic class, longer duration of treatment (1–3 years of treatment), and high cost of treatment amounting [24]. On the other hand, gender, educational qualification, socioeconomic status, and place of residence had shown no significant difference on QOL [13].

## 5. Conclusions

Families of children with childhood cancer face substantial challenges. The present study explored the QOL of the family and the impact of childhood cancer on these families. Results indicated that the domains of QOL are associated with the domains of impact on family. In addition, the domains of both tools were significantly associated with some demographic characteristics of the child and his parents. Based on these findings, increased psychosocial and emotional resources for patients and their families have to be facilitated and improved.

### 5.1. Implications

The result of this study revealed that the quality of life of the family of children diagnosed with cancer is affected in all aspects, psychologically, emotionally, socially, and financially. This implies that interventions should start as early as possible. Through the assessment, the patients and their families provide enough data for care plan. A multidisciplinary care plan to be formulated included all concerned healthcare professionals. Also, the results of these studies imply further research studies in this topic to investigate barriers and facilitators for family care.

## Figures and Tables

**Table 1:** Child demographic characteristics (*N* = 187).

	Mean	S.D.	Minimum	Maximum
Age	6.3 years	3.6	9 months	14 years

Gender	Male	88 (49.4%)		
	Female	90 (50.6%)		

Diagnosis	Leukemia	74 (41.6%)		
	Brain tumor	45 (25.3%)		
	Lymphomas	36 (20.2%)		
	Others	23 (12.9%)		

Time since diagnosis	2.0 years	1.4	6 months	8 years

Treatment phase	Completed	6 (3.4%)		
	Ongoing	154 (86.4%)		
	Not specified	16 (9%)		

Number of siblings	3.87	2.27	0	11

**Table 2:** Parents' demographic characteristics.

	Mother	Father
*Age*		
Mean	35.5	39.7
S.D.	7.58	8.23
Minimum	20	22
Maximum	50	58

*Marital status*		
Married	161 (90.4%)	159 (89.3%)
Divorced	17 (9.6%)	19 (10.7%)

*Educational level*		
General education	122 (68.5%)	89 (50%)
University	48 (27%)	77 (43.3%)
Postgraduate	8 (4.5%)	12 (12%)

*Employment status*		
Employed	137 (77%)	7 (3.9)
Unemployed	39 (21.9%)	159 (89.4%)
Retired	2 (1.1%)	12 (6.8%)

**Table 3:** Impact on family domains.

Domains	Mean	%
Familial social concerns	3.59	89.8
Financial burden	3.56	89.0
Mastery	3.43	85.8
Personal strain	3.21	80.3
Total of impact on family domains	3.45	

**Table 4:** Impact on the family subscales.

Statements	Mean	SD
*Financial burden (changes in the financial status of the family)*		
Additional income is needed in order to cover medical expenses	3.64	0.57
The illness is causing financial problems for the family	3.60	0.63
I am cutting down the hours I work to care for my child	3.55	0.75
Time is lost from work because of hospital appointments	3.54	0.68

*Familial/social concerns (level of disruption of interaction within the family and outside the family)*		
Because of the illness, we are not able to travel out of the city	3.70	0.56
We have little desire to go out because of my child's illness	3.66	0.61
Sometimes we have to change plans about going out at the last minute because of my child's state	3.66	0.60
Sometimes I wonder whether my child should be treated “specially” or the same as normal child	3.63	0.74
Don't have much time left over for other family members after caring for my child	3.61	0.72
I think about not having more children because of the illness	3.56	0.71
Our family gives up things because of my child's illness	3.54	0.76
We see family and friends less because of the illness	3.51	0.72
People in the neighborhood treat us specially because of my child's illness	3.48	0.73

*Personal strain (psychological burden experienced by care giver of the child with cancer)*		
Nobody understands the burden I carry	3.52	0.64
Sometimes I feel like we live on a roller coaster: in crisis when my child is acutely ill, OK when things are stable	3.49	0.66
I live from day to day and don't plan for the future	3.40	0.74
Fatigue is a problem for me because of my child's illness	3.31	0.74
It is hard to find a reliable person to take care of my child	3.19	0.76
Travelling to the hospital is a strain on me	2.40	0.65

*Mastery (coping strategy employed by the family)*		
Learning to manage my child's illness has made me feel better about myself	3.55	0.82
Because of what we have shared we are a closer family	3.48	0.90
We try to treat my child as if he/she were a normal child	3.43	0.89
My partner and I discuss my child's problem together	3.38	0.91
My relatives have been understanding and helpful with my child	3.32	0.86

**Table 5:** Family quality of life total.

Quality of life domains	Mean	%
Physical/material well-being	3.84	76.8
Family interaction	3.82	76.4
Emotional well-being	3.54	70.8
Parenting	3.53	70.6
Overall quality of life	3.68	

**Table 6:** Family quality of life subscale.

Quality of life domains	Mean	S.D.
*Physical/material well-being*		
My family feels safe at home, work, school, and in our neighborhood	4.29	0.93
My family gets medical care when needed	4.20	0.87
My family has a way to take care of our expenses	3.82	0.75
My family members have transportation to get to the places they need to be	3.68	0.85
My family gets dental care when needed	3.23	0.91

*Family interaction*		
My family is able to handle life's ups and downs	3.90	0.89
My family members talk openly with each other	3.87	0.79
My family solves problems together	3.86	0.81
My family members show that they love and care for each other	3.84	0.82
My family members support each other to accomplish goals	3.76	0.89
My family enjoys spending time together	3.73	0.73

*Emotional well-being*		
My family members have some time to pursue their own interests	3.61	0.95
My family has outside help available to us to take care of special needs of all family members	3.56	1.0
My family has the support we need to relieve stress	3.54	0.86
My family members have friends or others who provide support	3.47	0.92

*Parenting*		
Family members help the children learn to be independent	3.66	0.80
Family members teach the children how to get along with others	3.54	0.96
Family members help the children with schoolwork and activities	3.53	0.92
Adults in my family teach the children to make good decisions	3.51	1.01
Adults in my family know other people in the children's lives (friends, teachers)	3.51	1.01
Adults in my family have time to take care of the individual needs of every child	3.45	1.01

**Table 7:** Overall scores of impact on family domains correlation with demographic characteristics.

Variable	Mean ± SD	*P* value
*Gender*		
Male	3.5 ± 0.52	0.480
Female	3.4 ± 0.54	

*Diagnosis*		
Leukemia	3.6 ± 0.45	0.022
Brain tumor	3.4 ± 0.57	
Lymphomas	3.3 ± 0.49	
Others	3.4 ± 0.64	

*Phase of treatment*		
Completed	2.8 ± 0.13	0.001
Ongoing	3.5 ± 0.52	
Not specified	3.3 ± 0.56	

*Marital status (mother)*		
Married	3.5 ± 0.49	0.001
Divorced	2.7 ± 0.24	
Widow	—	

*Educational level (mother)*		
Uneducated	—	0.001
High school or less	3.6 ± 0.47	
University degree	3.3 ± 0.57	
Postgraduate	2.8 ± 0.38	

*Employment status (mother)*		
Unemployed	3.5 ± 0.47	0.003
Employed	3.2 ± 0.66	
Retired	2.8 ± 0.01	

*Marital status (father)*		
Married	3.5 ± 0.51	0.001
Divorced	2.9 ± 0.41	
Widow	—	

*Educational level (father)*		
Uneducated	—	0.001
High school or less	3.7 ± 0.32	
University degree	3.2 ± 0.61	
Postgraduate	3.1 ± 0.49	

*Employment status (father)*		
Unemployed	3.1 ± 0.56	0.001
Employed	3.5 ± 0.51	
Retired	2.8 ± 0.34	

**Table 8:** Overall scores of family's quality of life domains correlation with demographic characteristics.

Variable	Mean ± SD	*P* value
*Gender*		
Male	3.7 ± 0.58	0.248
Female	3.6 ± 0.53	

*Diagnosis*		
Leukemia	3.8 ± 0.58	0.076
Brain tumor	3.6 ± 0.59	
Lymphomas	3.7 ± 0.48	
Others	3.4 ± 0.48	

*Phase of treatment*		
Completed	4.0 ± 0.29	0.001
Ongoing	3.6 ± 0.55	
Not specified	4.1 ± 0.50	

*Marital status (mother)*		
Married	3.7 ± 0.52	0.390
Divorced	3.8 ± 0.84	
Widow	—	

*Educational level (mother)*		
Uneducated	—	0.462
High school or less	3.6 ± 0.53	
University degree	3.7 ± 0.59	
Postgraduate	3.9 ± 0.69	

*Employment status (mother)*		
Unemployed	3.6 ± 0.56	0.184
Employed	3.8 ± 0.55	
Retired	3.9 ± 0.01	

*Marital status (father)*		
Married	3.7 ± 0.52	0.596
Divorced	3.8 ± 0.79	
Widow	—	

*Educational level (father)*		
Uneducated	—	0.147
High school or less	3.7 ± 0.56	
University degree	3.6 ± 0.54	
Postgraduate	4.0 ± 0.56	

*Employment status (father)*		
Unemployed	4.2 ± 0.33	0.021
Employed	3.7 ± 0.56	
Retired	3.8 ± 0.29	

**Table 9:** Correlation between the overall scores of family's quality of life domains and the impact on family domains.

	Family interaction	Parenting	Emotional well-being	Physical/material well-being
Financial burden	0.162^*∗*^	−0.147	0.082	0.286^*∗*^
Familial/social concerns	0.120	−0.214^*∗*^	−0.069	0.286^*∗*^
Personal strain	0.97	−0.224^*∗*^	−0.084	0.304^*∗*^
Mastery	0.043	−0.208^*∗*^	−0.091	0.324^*∗*^

## Data Availability

The quantitative SPS data used to support the findings of this study are available from the corresponding author upon request.
